# Characteristics of COVID-19-associated multisystemic inflammatory syndrome in children treated in a Peruvian hospital, 2020-2022

**DOI:** 10.17843/rpmesp.2024.413.13736

**Published:** 2024-09-03

**Authors:** Noé Atamari-Anahui, Cynthia Huby-Muñoz, Claudia Peña-Coello, Deli Guillen-Buleje, Luis Gomez-Martinez, Héctor Nuñez-Paucar, Mariela Zamudio-Aquise, Raúl Bernal-Mancilla, Liz De Coll-Vela, Carlos Orellana-Siuce, Jorge Candela-Herrera

**Affiliations:** 1 Instituto Nacional de Salud del Niño-Breña, Lima, Peru. Instituto Nacional de Salud del Niño-Breña Lima Peru; 2 Research Unit for the Generation and Synthesis of Health Evidence, Vice Rectorate for Research, San Ignacio de Loyola University, Lima, Peru. San Ignacio de Loyola University Research Unit for the Generation and Synthesis of Health Evidence Vice Rectorate for Research San Ignacio de Loyola University Lima Peru; 3 San Martín de Porres University, Lima, Peru. San Martín de Porres University San Martín de Porres University Lima Peru; 4 Universidad Nacional Mayor de San Marcos, Lima, Peru. Universidad Nacional Mayor de San Marcos Universidad Nacional Mayor de San Marcos Lima Peru

**Keywords:** COVID-19, SARS-CoV-2, Multisystem Inflammatory Syndrome in Children, Kawasaki Disease, Intravenous Immunoglobulin

## Abstract

This study aimed to describe the characteristics of multisystemic inflammatory syndrome associated with COVID-19 (MIS-C) in the first three years of the pandemic in children in a pediatric hospital in Peru. We conducted an observational, descriptive study with data from 73 patients and described the clinical and laboratory characteristics, treatment and complications according to the wave of the pandemic and whether they had shock. The median age was 6 years, gastrointestinal and mucocutaneous manifestations were frequent in the three waves. Kawasaki disease-like phenotype was present in 34 (46.6%) patients and 21 (28.8%) patients developed shock. The most commonly used treatment was immunoglobulin (95.9%), followed by acetylsalicylic acid (94.5%) and corticosteroid (86.3%). Five (7%) patients had coronary aneurysm and 17 (23.3%) were admitted to the intensive care unit (ICU). Patients with shock had greater laboratorial alteration and need for mechanical ventilation. In conclusion, MIS-C has decreased in the first three years of the pandemic, possibly due to COVID-19 vaccination in children.

## INTRODUCTION

Multisystemic inflammatory syndrome in children (MIS-C) is a post-infectious complication of SARS-CoV-2 ^1^, which appeared weeks after the peak of COVID-19 infections in different countries ^2^^-^^6^. It is a rare disease with an incidence of 4.9 cases/million person-months in children under five years of age and 6.3 cases/million person-months in children aged six to ten years ^2^.

MIS-C appears lately (>5 years), and its representative manifestations are gastrointestinal such as abdominal pain and mucocutaneous such as conjunctival injection and polymorphous exanthema ^7^. For this reason, it has similar symptomatology to Kawasaki disease (KD), toxic shock syndrome or hemophagocytic lymphohistiocytosis ^1^^,^^8^. The most frequent complications are cardiovascular which are the main reasons for admission to pediatric intensive care units (PICU) ^9^^,^^10^. The clinical-epidemiological characteristics of MIS-C varied during the pandemic ^4^^,^^11^. In France, children with MIS-C had less severe disease throughout the waves of COVID-19 ^12^, similar to India ^13^^)^ and Argentina ^14^. This was probably related to the diagnosis and treatment that each country was able to implement according to its available resources ^12^^,^^13^^,^^15^^)^ and by vaccination against SARS-CoV-2 and by the type of variant that predominated in each wave of the pandemic ^5^.

Due to the epidemiological change of MIS-C in recent years ^4^, it is important to understand how the characteristics of MIS-C have varied during the pandemic in Peru, in order to improve care protocols for future scenarios and avoid complications in patients. This study aimed to describe the characteristics of MIS-C during the first three years of the pandemic in a pediatric hospital in Peru according to the wave and the development of shock.

**KEY MESSAGES**

Motivation for the study. There are few studies describing the variation of COVID-19-associated multisystem inflammatory syndrome (MIS-C) in Peru across pandemic waves.

Main findings. Cases of MIS-C decreased during the first three years of the pandemic, with higher frequency in the second wave with clinical features similar to Kawasaki disease.

Implications. MIS-C is a post-infectious complication of SARS-CoV-2. Its diagnostic suspicion is important weeks after peak infections, especially in children who have not yet received COVID-19 vaccines.

## THE STUDY

We conducted an observational, descriptive and retrospective study. The study population consisted of hospitalized patients diagnosed with MIS-C between April 2020 and December 2022 at the Instituto Nacional de Salud del Niño-Breña (INSN-B) in Lima, Peru. INSN-B is one of the pediatric hospitals with the highest resolution capacity located in Lima, with different specialties and subspecialties, including the pediatric intensive care unit (PICU) for critical patients. The INSN-B is a state institution that belongs to the Peruvian Ministry of Health (MINSA) and serves the population with or without comprehensive health insurance (SIS) and is one of the most representative institutions in Peru, both in terms of care and research ^16^.

MIS-C was diagnosed based on World Health Organization (WHO) criteria ^1^, similar to previous reports ^12^^,^^13^. Patients with diagnosis of MIS-C at emergency admission or during hospitalization were included. We excluded patients with SARS-CoV-2 infection and KD criteria without MIS-C criteria (three patients), those diagnosed with another disease (one with thrombotic thrombocytopenic purpura, one with Takayasu arteritis and two with dengue) and those referred to another hospital (three patients).

We did not calculate sample size due to the limited number of cases since this is a rare disease. Data collection was performed by four researchers distributed in two independent groups. Discrepancies were resolved by a fifth researcher performing the review again.

### Study variables

The study variables were age (years), sex (male and female), intradomiciliary contact with SARS-COV-2 infected person (yes or no), type of SARS-CoV-2 test: real-time polymerase chain reaction (RT-PCR), antigenic and serologic (IgM and IgG), comorbidities, time of illness (days), gastrointestinal symptomatology (abdominal pain, vomiting, diarrhea, nausea), mucocutaneous (polymorphous exanthema, conjunctival injection, changes in the oral cavity, edema of palms and soles, desquamation of fingers and cervical lymphadenopathy), respiratory (upper respiratory symptoms, respiratory distress), neurological and clinical phenotype (fever and inflammation, similar to KD and shock), based on previous reports ^6^^,^^17^^,^^18^.

We also considered laboratory characteristics, treatment (immunoglobulin, corticosteroid, acetylsalicylic acid, vasopressors/inotropes, anticoagulation and antibiotics), complications (echocardiographic alterations, admission to PICU and invasive mechanical ventilation, macrophage activation syndrome) and hospitalization time (days) (Table S1).

The variables were described in a general way (period 2020-2022), according to the first three waves by COVID-19 (first: March - December 2020, second: January - September 2021, third: December 2021 - January 2022) ^19^ and according to the shock phenotype (MIS-C with need of support with inotrope/vasopressor or fluid resuscitation >20 ml/kg).

### Procedure and statistical analysis

We identified the records of the patients included in the study and then we proceeded to review and collect the information from their medical records in the hospital archive. A data collection form was used, after approval and authorization of the project by the hospital’s institutional research committee.

The STATA v.16 program (StataCorp LP, College Station, Texas, United States) was used for data analysis. Categorical variables were described by absolute and relative frequencies and quantitative variables by mean and standard deviation (SD) or median and interquartile range (IQR) according to the normality assessment with the Shapiro-Wilk test.

### Ethical aspects

The study was approved by the INSN-B Research Ethics Committee (No. 078-2022-CIEI-INSN) and the protocol was registered in the PRISA platform of the National Institute of Health (code: EI00002522). Informed consent was not requested because we opted for the permission of the institution for the collection of data from the medical records; we respected the confidentiality of the data by using numerical codes. Some characteristics of the first eight patients in the study were published in the Revista Peruana de Medicina Experimental y Salud Pública ^20^.

## FINDINGS

**Figure 1:**
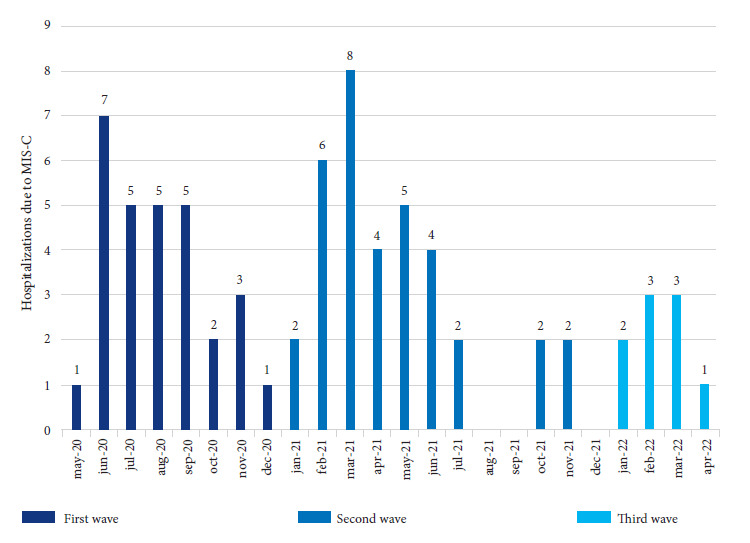
Hospitalizations due to COVID-19-associated multisystemic inflammatory syndrome (MIS-C) according to pandemic waves (first wave: March-December 2020, second wave: January-September 2021, third wave: December 2021-January 2022).

Of the 73 patients with MIS-C, about 50% were diagnosed during the second wave (Figure 1) and the last patient was diagnosed in April 2022 (third wave). The median age was 6 years (IQR: 2 - 9, range: 3 months - 16 years) and was higher in the second and third waves. Five participants (6.9%) were less than one year old, 23 (31.5%) one to four years, 32 (43.8%) five to nine years and 13 (17.8%) older than 10 years. More than half were male and 61 (83.6%) patients had intradomiciliary contact with a person infected with SARS-COV-2. Sixty-one (83.6%) had any positive SARS-CoV-2 test (6/59 RT-PCR, 3/49 antigenic, 17/56 IgM and 52/64 IgG) and 12 had negative tests with positive intradomiciliary contact to SARS-CoV-2. Two patients had comorbidities (one neurogenic bladder and one unilateral hydronephrosis). The median time of illness was 5 days (IQR: 4-7) and was longer in the third wave (Table 1).

**Table 1:** General and clinical characteristics of patients with multisystem inflammatory syndrome associated with COVID-19 (MIS-C).

Characteristics		Total n=73 (%)	Pandemic waves			Shock	
			First wave n=29 (%)	Second wave n=35 (%)	Third wave n=9 (%)	No n=52 (%)	Yes n=21 (%)
Age (years) ^a^		6 (2-9)	5 (2-7)	6 (2-9)	9 (1.6-13.8)	5 (2-8.5)	8 (5-9)
Male sex		46 (63.0)	19 (65.5)	20 (57.1)	7 (77.8)	33 (63.5)	13 (61.9)
Intradomiciliary contact		61 (83.6)	22 (78.9)	31 (88.6)	8 (88.9)	42 (80.8)	19 (90.5)
Time of illness (days) ^a^		5 (4-7)	5 (4-7)	5 (4-7)	6 (5-8)	5,5 (4-7)	5 (4-5)
Clinical manifestations							
	Fever time (days) ^a^	5 (4-7)	4 (4-6)	5 (4-7)	6 (5-8)	5 (4-7)	5 (4-6)
	Gastrointestinal ^b^	60 (82.2)	23 (79.3)	29 (82.9)	8 (88.9)	42 (80.8)	18 (85.7)
	Abdominal pain	41 (56.2)	16 (55.2)	22 (62.9)	3 (33.3)	25 (48.1)	16 (76.2)
	Vomiting	32 (43.8)	12 (41.3)	15 (42.9)	5 (55.6)	18 (40.0)	12 (57.1)
	Diarrhea	30 (41.1)	10 (34.5)	16 (45.7)	4 (44.4)	22 (42.3)	8 (38.1)
	Nausea	12 (16.4)	4 (13.8)	5 (14.3)	3 (33.3)	7 (13.5)	5 (23.8)
	Mucocutaneus ^b^	66 (90.4)	26 (89.7)	31 (88.6)	9 (100.0)	47 (90.4)	19 (90.5)
	Polymorphous exanthem	55 (75.3)	21 (72.4)	26 (74.3)	8 (88.9)	40 (76.9)	15 (71.4)
	Conjunctival injection	48 (65.8)	20 (69.0)	22 (62.9)	6 (66.7)	32 (61.5)	16 (76.2)
	Changes in lips/tongue/oral mucosa	25 (34.3)	6 (20.7)	13 (37.1)	6 (66.7)	19 (36.5)	6 (28.6)
	Edema of palms and soles	23 (31.5)	7 (24.1)	12 (34.3)	4 (44.4)	18 (34.6)	5 (23.8)
	Skin peeling on fingertips	5 (6.9)	0 (0.0)	4 (11.4)	1 (11.1)	2 (3.9)	3 (14.3)
	Cervical lymphadenopathies >1.5 cm	13 (17.8)	4 (13.8)	7 (20.0)	2 (22.2)	7 (13.5)	6 (28.6)
	Respiratory	21 (28.8)	6 (20.7)	11 (31.4)	4 (44.4)	14 (26.9)	7 (33.3)
	High respiratory symptoms (cough, coryza, odynophagia)	14 (19.2)	4 (13.8)	7 (20.0)	3 (33.3)	12 (23.1)	2 (9.5)
	Respiratory distress (tachypnea, tugging, wheezing)	9 (12.3)	2 (6.9)	5 (14.3)	2 (22.2)	3 (5.8)	6 (28.6)
	Neurological (headache, irritability, seizures)	14 (19.2)	3 (10.3)	9 (25.7)	2 (22.2)	6 (11.5)	8 (38.1)
Clinical phenotype							
	Fever and inflammation	18 (24.6)	5 (17.2)	9 (25.7)	4 (44.4)	--	--
	Similar to Kawasaki disease	34 (46.6)	16 (55.2)	14 (40.0)	4 (44.4)	--	--
	Shock	21 (28.8)	8 (27.6)	12 (34.3)	1 (11.1)	--	--

a Median and interquartile ranges, ^b^ presented more than one symptom. Shock: need for inotropic support or resuscitation with fluids >20 mL/kg.

Gastrointestinal (abdominal pain and vomiting) and mucocutaneous (polymorphous exanthema and conjunctival injection) manifestations were frequent during all three waves, while respiratory and neurological manifestations were frequent during the second wave. The KD-like phenotype was frequent in all three waves and was reported in 34 (46.6%) patients followed by the shock phenotype in 21 (28.8%) patients.

Blood tests showed mild anemia: 10.5 g/dL (IQR: 9.7-11.2), and elevated leukocytes: 12,500/mm^3^ (IQR: 7330-16,390), neutrophils: 8772/mm^3^ (IQR: 5057.7-12,002), erythrocyte: sedimentation rate: 39.5 mm/h (IQR: 27.5-49), C-reactive protein: 18.2 mg/dL (IQR: 11.8-24.8), liver enzymes, fibrinogen: 558 mg/dL (IQR: 493.3-651.5), D-dimer: 4.2 mg/L (IQR: 2.3-7.1), ferritin: 402 ng/mL (IQR: 234.5-538.5) and lactate dehydrogenase: 547 U/L (IQR: 432-665) (Table 2).

**Table 2:** Laboratory characteristics of patients with multisystem inflammatory syndrome associated with COVID-19 (MIS-C).

Laboratory Features	Total n=73	Shock	
		No n=52	Yes n=21
	Median (IQR)	Median (IQR)	Median (IQR)
Hemoglobin (g/dL)	10.5 (9.7-11.2)	10.7 (9.7-11.2)	10.2 (9.7-11.4)
Leukocytes (cel/mm^3^)	12,500 (7330-16390)	13,170 (8315-16420)	10,260 (6100-14,760)
Neutrophiles (cel/mm^3^)	8772 (5057.7-12,002)	9618.8 (4975-12,334.9)	8174 (5322.2-11,219)
Lymphocytes (cel/mm^3^)	1979.1 (893.6-3452)	2332.6 (1073.1-4461.3)	1405.2 (542.3-2902.6)
Eosinophiles (cel/mm^3^)	73.3 (0-285.4)	95.4 (0-286.6)	59.8 (35-237.7)
Platelets (cel/mm^3^)	17,400 (110,000-323,000)	196,000 (116,000-369,500)	138,000 (100,000-252,000)
Erythrocyte sedimentation rate (mm/h)	39.5 (27.5-49)	40 (25-50)	38.5 (32-45)
C-reactive protein (mg/dL)	18.2 (11.8-24.8)	17.7 (10.9-24.5)	18.5 (13.6-27.3)
Aspartate aminotransferase (UI/L)	44 (34-67)	43 (33-74)	44 (42-49)
Alanine aminotransferase (UI/L)	47.5 (23-77)	43 (20-77)	50.5 (27-120)
Gamma-glutamyltransferase (UI/L)	54.2 (27-176.5)	38 (19-86)	175 (59-301)
Alkaline phosphatase (UI/L)	415 (306.5-504.5)	375.5 (286-466.5)	458.5 (405.5-623.5)
Fibrinogen (mg/dL)	558 (493.3-651.5)	558 (509.8-658.5)	557 (492.2-643.4)
D-dimer (mg/L)	4.2 (2.3-7.1)	3.9 (2.3-7.2)	4.5 (2.4-6.9)
Albumin (g/dL)	3.7 (3.3-4)	3.7 (3.4-3.9)	3.4 (3.1-4)
Sodium (mmol/L)	135 (132-137)	134 (131-136)	136.5 (132.5-138.5)
Potassium (mmol/L)	3.9 (3.6-4.3)	3.9 (3.7-4.4)	3.9 (3.6-4.2)
Ferritin (ng/mL)	402 (234.5-538.5)	359 (217-529)	436 (370-649)
CPK-MB (UI/L)	20 (15.5-43)	21.5 (13.5-29.5)	19.5 (17-29)
Lactate dehydrogenase (UI/L)	547 (432-665)	560 (458-737)	462 (426-644)
Urea (mg/dL)	27 (19-40)	23 (18-40)	34 (21-40)
Creatinine (mg/dL)	0.5 (0.4-0.6)	0.5 (0.4-0.6)	0.5 (0.4-0.5)

IQR: interquartile range; CPK-MB: myocardial creatinine phosphokinase; shock: need for inotropic support or fluid resuscitation >20mL/kg.

Intravenous human immunoglobulin was used in 70 (95.9%) patients with a dose of 1 to 2gr/kg (minimum: 9 grams; maximum: 100 grams) and was administered to all patients during the first wave. Corticosteroids (methylprednisolone) were more frequently used during the second wave in 34 (97.1%) patients. The dose was 1-2mg/kg for five days, and 10-30mg/kg as a pulse dose (14 patients) in those with signs of clinical deterioration for three days. Acetylsalicylic acid was used in 69 (94.5%) patients, 47 (64.4%) received prophylactic anticoagulation and 57 (78.1%) antibiotics, the latter two decreased their use during each wave.

Regarding complications, five developed coronary aneurysm and three developed left ventricular dysfunction. Two patients required intubation in the emergency room, 17 (23.3%) were admitted to PICU (mean 5.8+/-3 days) and 11 (15.1%) required invasive mechanical ventilation (mean 4+/-1 day). Ten (15.1%) patients had macrophage activation syndrome, six (8.2%) had pneumonia, two had pancreatitis and one had deep vein thrombosis of the extremities. The median length of hospitalization was 10 days (IQR: 8-14) and no patient died during hospitalization.

Patients with the shock phenotype were older with a median age of eight years (IQR: 5-9), had greater respiratory distress and neurological symptoms compared to those who did not develop shock (Table 1). In addition, greater inflammatory alterations (increased c-reactive protein and ferritin), alteration of liver enzymes, hematological alterations (lower hemoglobin, lymphopenia and mild thrombocytopenia) and coagulation alterations (fibrinogen and D-dimer) were found in this group of patients (Table 2). The use of second dose immunoglobulin was higher (47.6%) and all used vasopressors and antibiotics (Table 3). Echocardiographic alterations were more frequent in these patients and they had longer hospitalization time, 14 days (IQR: 11-16).

**Table 3:** Treatment and complications in patients with multisystem inflammatory syndrome associated with COVID-19 (MIS-C).

Characteristics			Total n=73 (%)	COVID-19 wave			Shock	
				1^st^ wave n=29 (%)	2^nd^ wave n=35 (%)	3^rd^ wave n=9 (%)	No n=52 (%)	Yes n=21 (%)
Treatment								
		Human immunoglobulin (1st dose)	70 (95.9)	29 (100.0)	33 (94.3)	8 (88.9)	49 (94.2)	21 (100.0)
		Human immunoglobulin (2nd dose)	19 (26.0)	7 (24.1)	11 (31.4)	1 (11.1)	9 (17.3)	10 (47.6)
		Corticoids	63 (86.3)	21 (72.4)	34 (97.1)	8 (88.9)	44 (84.6)	19 (90.5)
		Acetylsalicylic acid	69 (94.5)	27 (93.1)	34 (97.1)	8 (88.9)	50 (96.2)	19 (90.5)
		Vasopressors/inotropes	21 (28.8)	8 (27.6)	12 (34.3)	1 (11.1)	0 (0.0)	21 (100.0)
		Anticoagulation						
		Treatment	5 (6.9)	2 (6.9)	2 (5.7)	1 (11.1)	1 (1.9)	4 (19.1)
		Prophylactic	47 (64.4)	20 (69.0)	23 (65.7)	4 (44.4)	32 (61.5)	15 (71.4)
		Antibiotics	57 (78.1)	24 (82.8)	28 (80.0)	5 (55.6)	36 (69.2)	21 (100.0)
Complications								
	Echocardiography (n=71)^a^							
		Coronary aneurysm	5 (7.0)	1 (3.6)	3 (8.6)	1 (12.5)	3 (5.9)	2 (10.0)
		Left ventricular dysfunction	3 (4.2)	2 (7.1)	1 (2.9)	0 (0.0)	1 (2.0)	2 (10.0)
		Pericardial effusion	7 (9.9)	5 (17.9)	1 (2.9)	1 (12.5)	3 (5.9)	4 (20.0)
		Valvular alteration	4 (5.6)	3 (10.7)	1 (2.9)	0 (0.0)	1 (2.0)	3 (15.0)
		Ventricular dilatation	1 (1.4)	1 (3.6)	0 (0.0)	0 (0.0)	0 (0.0)	1 (5.0)
	Admission to PICU		17 (23.3)	7 (24.1)	9 (25.7)	1 (11.1)	0 (0.0)	17 (80.9)
	Invasive mechanical ventilation		11 (15.1)	4 (13.8)	6 (17.1)	1 (11.1)	0 (0.0)	11 (52.4)
	Macrophage activation syndrome		10 (15.1)	4 (13.8)	5 (14.3)	2 (22.2)	8 (15.4)	3 (14.3)
	Pneumonia		6 (8.2)	2 (6.9)	4 (11.4)	0 (0.0)	3 (5.8)	3 (14.3)
	Time of hospitalization (days) ^b^		10 (8-14)	10 (7-13)	10 (8-14)	8 (8-10)	8.5 (7-11)	14 (11-16)

a They had more than one finding, ^b^ median (IQR).

IQR: interquartile range, PICU: Pediatric Intensive Care Unit, shock: need for inotropic support or resuscitation with fluids > 20mL/kg.

## DISCUSSION

Our study showed that the frequency of MIS-C varied during the first three years of the pandemic and was characterized by gastrointestinal and mucocutaneous symptoms. Treatment mainly consisted of immunoglobulin and corticosteroids, and those with shock had more complications.

We found that more cases were diagnosed during the second wave, which is similar to what was reported in a hospital in India ^13^, but a study in France reported more cases during the first wave ^12^. This difference could be explained by the fact that the high peak of infection in Peru occurred during the second wave in addition to the softening of social distancing measures due to economic recovery and increased movement of people, including children ^19^. MIS-C has been reported four to six weeks after the peak of COVID-19 infection ^1^, which is related to what was reported in France during the first wave ^12^.

Variants may also influence the frequency of MIS-C cases. In Peru, the identification of variants began during the second wave (2021) with the Lambda/Gamma/Delta variant predominating; the Omicron variant was the most prevalent during the third wave, ^19^ where fewer MIS-C cases were recorded despite a high peak of infections. Vaccination may have influenced the decrease in MIS-C cases during the third wave. The effect of vaccination was reported during the wave for the Delta variant in children aged 12-18 years in the United States ^21^, and during the wave for the Omicron variant in Denmark ^5^.

In Peru, vaccination (Pfizer-BioNTech) started in children aged 12-17 years in November 2021 and 5-11 years in January 2022. In our study, two patients with MIS-C had received vaccination against COVID-19, a 14-year-old male (vaccinated in November 2021, one dose) who developed disease five months later (he was IgG positive), and an 11-year-old male (vaccinated in February 2022) who developed the disease eight days later, but had positive IgG and IgM serology at diagnosis.

The median age was six years, lower than that reported by other studies ^9^^,^^18^^)^ and higher in later waves, a trend similar to that reported in Argentina ^14^. Gastrointestinal and mucocutaneous manifestations were the most frequent, in agreement with other studies ^12^^,^^13^. These characteristics were reported since the first cases in the United Kingdom ^6^, and are also found in reports from Denmark ^5^, United States ^11^^)^ and Latin America ^3^^,^^10^.

Children with MIS-C developed laboratory alterations such as elevation of inflammatory markers, hematological and coagulation disorders, particularly in patients with the shock phenotype in agreement with previous reports ^9^^,^^18^. These alterations are produced by an imbalance in the immune system due to the binding of SARS-CoV-2 to ACE-2 receptors in the endothelial cells of the vascular system, causing inflammation and systemic coagulation ^1^.

Treatment consisted in the use of immunoglobulin, corticoids and acetylsalicylic acid, which is similar to the management of KD ^22^^)^ and according to the management of MIS-C in Latin American countries such as Chile ^17^ and Argentina ^15^. The patients that required a second dose of immunoglobulin did not show clinical improvement 36 hours after the first dose, they had persistent fever or developed shock or macrophage activation syndrome.

The use of antibiotics was high at the beginning of the pandemic; patients with severe MIS-C or COVID-19 who were most at risk of receiving such treatment ^18^^,^^20^. In our study, antibiotics were used more during the first and second waves because of the risk of bacterial infection; however, it was lower in the third wave, a trend similar to that reported in France ^12^. This is explained by the fact that as the waves passed, diagnosis and treatment improved, avoiding the use of antibiotics, since MIS-C is a post-infectious viral complication.

Cardiac complications characterized MIS-C during the initial phase ^23^^,^^24^. In our study, five (7.0%) patients developed coronary aneurysm and three (4.2%) left ventricular dysfunction, none had coronary artery dilatation. This low frequency may be explained by the fact that there were fewer severe cases resulting in less admission to PICU and need for mechanical ventilation in contrast to other studies ^3^^,^^12^^,^^13^^,^^23^^,^^24^; however, patients with shock had more cardiac alterations and complications similar to other reports ^9^^,^^11^^,^^18^^,^^24^. No patient died during hospitalization but other studies have reported a lethality rate between 1 to 9% ^9^^,^^13^^,^^14^^,^^18^.

The study was limited by the fact that it was retrospective and was conducted in a single hospital; therefore, these findings cannot be generalized to other institutions in Peru. It was not possible to identify the type of SARS-CoV-2 variant in each patient, which would have served to better characterize each group; however, the cases were temporally related to the COVID-19 waves. In spite of this, this study has the strength of describing characteristics of a considerable series of MIS-C cases from a national pediatric referral hospital during the first three years of the pandemic, since the Peruvian health system had limited human, material and logistic resources.

In conclusion, the frequency of MIS-C cases in INSN-B varied during the first three years of the pandemic. The clinical manifestations and treatment were similar to those described by other studies, and patients with shock had greater laboratory alterations and cardiac complications.
