# Supplementary material for: Characteristics of COVID-19-associated multisystemic inflammatory syndrome in children treated in a Peruvian hospital, 2020-2022
Source: Rev Peru Med Exp Salud Publica. 2024 Sep 3;41(3):301–8. doi: 10.17843/rpmesp.2024.413.13736 (PMC11495924; doi:10.17843/rpmesp.2024.413.13736)
Supplement: Supplementary material. — Available in the electronic version of the RPMESP. [file rpmesp-41-03-13736-s001.docx]

**Material suplementario**

**Tabla S1.** Definición operacional de las variables utilizadas en el estudio

| Variables | Definición operacional | Categorías | Unidad de medida |
| --- | --- | --- | --- |
| Edad | Diferencia entre la fecha de diagnóstico y fecha de nacimiento | -- | Años |
| Sexo | Sexo consignado en la historia clínica | - Masculino - femenino | -- |
| Contacto intradomiciliario | Reporte de persona positivo a SARS-COV-2 dentro de la vivienda del paciente | - No - Si | -- |
| Prueba SARS-COV-2 | Tipo de prueba para detectar SARS-COV-2 | - Reacción en cadena de polimerasa en tiempo real (RT-PCR) - Antigénica - Serológicas (IgM/IgG) | -- |
| Comorbilidades | Enfermedad diagnosticada antes o durante la enfermedad actual | - No - Si | -- |
| Tiempo de enfermedad | Tiempo transcurrido entre el inicio de los signos y síntomas hasta el diagnóstico | -- | Dias |
| Manifestaciones clínicas |  |  |  |
| Tiempo de fiebre (días)* | Tiempo de duración de fiebre | -- | Dias |
| Gastrointestinales |  |  |  |
| Dolor abdominal | Registro de dolor en alguna región del abdomen | - No - Si | -- |
| Vómitos | Registro de la expulsión del contenido del estómago por la boca | - No - Si | -- |
| Diarrea | Registro de cambio en la consistencia o frecuencia de las deposiciones | - No - Si | -- |
| Náuseas | Registro de sensación o necesidad de vomitar | - No - Si | -- |
| Mucocutáneas |  |  |  |
| Exantema polimorfo | Registro de lesiones rojas distribuidas en la piel | - No - Si | -- |
| Inyección conjuntival | Registro de coloración roja de los ojos (parte blanca del ojo) | - No - Si | -- |
| Cambios en la cavidad oral | Registro de labios agrietados, labios rojos, lengua papilada o en “fresa”, mucosa oral roja. | - No - Si | -- |
| Edema de palmas y plantas | Registro de aumento de volumen en dorso o palma de manos y pies. | - No - Si | -- |
| Descamación de los pulpejos de los dedos | Registro de descamación en la extremidad distal de los dedos | - No - Si | -- |
| Linfoadenopatías cervicales >1,5cm | Registro de tumoración cervical palpable >1,5cm (unilateral o bilateral) | - No - Si | -- |
| Respiratorias |  |  |  |
| Síntomas respiratorios altos | Registro de síntomas respiratorios altos como tos, coriza, odinofagia. | - No - Si | -- |
| Dificultad respiratoria | Registro de dificultad respiratoria caracterizado por taquipnea, tiraje o quejido. | - No - Si | -- |
| Neurológicas | Registro de sintomatología neurológica como cefalea, irritabilidad o convulsiones. | - No - Si | -- |
| Fenotipo clínico |  |  |  |
| Fiebre e inflamación | SIM-C que no tenía criterios de shock o de EK, y que se encontraba hemodinámicamente estable. | - No - Si | -- |
| Similar a enfermedad de Kawasaki | SIM-C que tenía criterios de EK completo o incompleto según la “*American Heart Association*”, pero sin shock. | - No - Si | -- |
| Shock | SIM-C con necesidad de soporte con inotrópico o vasopresor o reanimación de fluidos >20 ml/kg. | - No - Si | -- |
| Laboratorios |  |  |  |
| Hemoglobina | Valor obtenido del registro de laboratorio. |  | g/dL |
| Leucocitos | Valor obtenido del registro de laboratorio. |  | cél/mm^3^ |
| Neutrófilos | Valor obtenido del registro de laboratorio. |  | cél/mm^3^ |
| Linfocitos | Valor obtenido del registro de laboratorio. |  | cél/mm^3^ |
| Eosinófilos | Valor obtenido del registro de laboratorio. |  | cél/mm^3^ |
| Plaquetas | Valor obtenido del registro de laboratorio. |  | cél/mm^3^ |
| Velocidad de sedimentación globular | Valor obtenido del registro de laboratorio. |  | mm/h |
| Proteína c reactiva | Valor obtenido del registro de laboratorio. |  | mg/dL |
| Aspartato aminotransferasa | Valor obtenido del registro de laboratorio. |  | UI/L |
| Alanino aminotransferasa | Valor obtenido del registro de laboratorio. |  | UI/L |
| Gamma-glutamiltransferasa | Valor obtenido del registro de laboratorio. |  | UI/L |
| Fosfatasa alcalina | Valor obtenido del registro de laboratorio. |  | UI/L |
| Fibrinógeno | Valor obtenido del registro de laboratorio. |  | mg/dL |
| Dímero D | Valor obtenido del registro de laboratorio. |  | mg/L |
| Albúmina | Valor obtenido del registro de laboratorio. |  | g/dL |
| Sodio | Valor obtenido del registro de laboratorio. |  | mmol/L |
| Potasio | Valor obtenido del registro de laboratorio. |  | mmol/L |
| Ferritina | Valor obtenido del registro de laboratorio. |  | ng/mL |
| CPK-MB | Valor obtenido del registro de laboratorio. |  | UI/L |
| Lactato deshidrogenasa | Valor obtenido del registro de laboratorio. |  | UI/L |
| Urea | Valor obtenido del registro de laboratorio. |  | mg/dL |
| Creatinina | Valor obtenido del registro de laboratorio. |  | mg/dL |
| Tratamiento |  |  |  |
| Inmunoglobulina humana (1era dosis) | Registro de infusión de inmunoglobulina humana | - No - Si | -- |
| Inmunoglobulina humana (2da dosis) | Registro de segunda infusión de inmunoglobulina humana | - No - Si | -- |
| Corticoide | Registro de uso de corticoide endovenoso (metilprednisolona, dexametasona, hidrocortisona) | - No - Si | -- |
| Ácido acetil salicílico | Registro de uso de ácido acetil salicílico | - No - Si | -- |
| Vasopresores/inotrópicos | Registro de uso de medicamentos como adrenalina, noradrenalina, dopamina, dobutamina, entre otros. | - No - Si | -- |
| Anticoagulación |  |  |  |
| Tratamiento | Registro de uso de anticoagulante de bajo peso molecular (enoxaparina) dosis de tratamiento | - No - Si | -- |
| Profiláctico | Registro de uso de anticoagulante de bajo peso molecular (enoxaparina) dosis de profilaxis | - No - Si | -- |
| Antibióticos | Registro de uso de antibiótico durante la hospitalización | - No - Si | -- |
| Complicaciones |  |  |  |
| Aneurisma coronario | Registro de arterias coronarias con valor Z score (≥ 2.5) en el ecocardiografía | - No - Si | -- |
| Ingreso a UCIP | Registro de ingreso a la Unidad de Cuidados Intensivos Pediátricos (UCIP) | - No - Si | -- |
| Ventilación mecánica invasiva | Registro de uso de ventilador mecánico como soporte respiratório | - No - Si | -- |
| Síndrome de activación de macrófagos | Síndrome caracterizado por ferritina > 684 ng/mL más dos de los siguientes: plaquetas ≤181 000/mm^3^, aspartato aminotrasnferasa (AST) >48 U/L, triglicéridos ≥ 156 mg/dL y fibrinógeno ≤360 mg/dL | - No - Si | -- |
| Neumonía | Registro de diagnóstico de neumonía al diagnóstico de SIM-C o durante la hospitalización | - No - Si |  |
| Tiempo de hospitalización (días)* | Diferencia entre la fecha de egreso y la fecha de ingreso | -- | Dias |

SIM-C: Síndrome Inflamatorio Multisistémico asociado a COVID-19. EK: Enfermedad de Kawasaki
