# Supplementary material for: Characteristics of COVID-19-associated multisystemic inflammatory syndrome in children treated in a Peruvian hospital, 2020-2022
Source: Rev Peru Med Exp Salud Publica. 2024 Sep 3;41(3):301–8. doi: 10.17843/rpmesp.2024.413.13736 (PMC11495924; doi:10.17843/rpmesp.2024.413.13736)
Supplement: Translation

# Translation: Características del síndrome inflamatorio multisistémico asociado a COVID-19 en niños atendidos en un hospital peruano, 2020-2022

**Authors:** Noé Atamari-Anahui, Cynthia Huby-Muñoz, Claudia Peña-Coello, Deli Guillen-Buleje, Luis Gomez-Martinez, Héctor Nuñez-Paucar, Mariela Zamudio-Aquise, Raúl Bernal-Mancilla, Liz De Coll-Vela, Carlos Orellana-Siuce, Jorge Candela-Herrera

**Affiliations:** 1 Instituto Nacional de Salud del Niño-Breña, Lima, Perú.; 2 Unidad de Investigación para la Generación y Síntesis de Evidencias en Salud, Vicerrectorado de Investigación, Universidad San Ignacio de Loyola, Lima, Perú.; 3 Universidad San Martín de Porres, Lima, Perú.; 4 Universidad Nacional Mayor de San Marcos, Lima, Perú.

**Keywords:** COVID-19, SARS-COV-2, Síndrome Inflamatorio Multisistémico en Niños, Enfermedad de Kawasaki, Inmunoglobulina Endovenosa

## Abstract

El propósito del estudio fue describir las características del síndrome inflamatorio multisistémico asociado a COVID-19 (SIM-C) en los primeros tres años de pandemia en niños de un hospital pediátrico del Perú. Se realizó un estudio observacional, descriptivo con datos de 73 pacientes y se describieron las características clínicas, laboratoriales, tratamiento y complicaciones según la ola de la pandemia y si tuvieron shock. La mediana de edad fue 6 años, las manifestaciones gastrointestinales y mucocutáneas fueron frecuentes en las tres olas. El fenotipo similar a enfermedad de Kawasaki se presentó en 34 (46,6%) pacientes y 21 (28,8%) pacientes desarrollaron shock. El tratamiento más usado fue la inmunoglobulina (95,9%), ácido acetil salicílico (94,5%) y corticoide (86,3%). Cinco (7%) pacientes tuvieron aneurisma coronario y 17 (23,3%) ingresaron a la unidad de cuidados intensivos (UCI). Los pacientes con shock tuvieron mayor alteración laboratorial y necesidad de ventilación mecánica. En conclusión, el SIM-C ha disminuido en los primeros tres años de pandemia posiblemente por la vacunación de COVID-19 en niños.

## INTRODUCCIÓN

El síndrome inflamatorio multisistémico en niños (SIM-C) es una complicación posinfecciosa del SARS-CoV-2 ^1^, que se presentó semanas después del pico de contagios de COVID-19 en diferentes países ^2^^-^^6^. Es una enfermedad poco frecuente con una incidencia de 4,9 casos/millón personas-mes en menores de cinco años y 6,3 casos/millón de personas-mes en niños de seis a diez años ^2^.

En el SIM-C, la edad de presentación es más tardía (>5 años), las manifestaciones gastrointestinales como el dolor abdominal y mucocutáneas como la inyección conjuntival y el exantema polimorfo son representativas ^7^. Por ese motivo comparte sintomatología similar a la enfermedad de Kawasaki (EK), síndrome de shock tóxico o linfohistiocitosis hemofagocítica ^1^^,^^8^. Las complicaciones más frecuentes son las cardiovasculares y es el principal motivo de ingreso a las unidades de cuidados intensivos pediátricos (UCIP) ^9^^,^^10^. Las características clínico-epidemiológicas del SIM-C fueron variando durante la pandemia ^4^^,^^11^. En Francia, los niños con SIM-C tuvieron enfermedad menos grave a lo largo de las olas de la COVID-19 ^12^, similar a India ^13^^)^ y Argentina ^14^. Esto probablemente relacionado al diagnóstico y tratamiento que cada país pudo implementar según sus recursos disponibles ^12^^,^^13^^,^^15^ y por la vacunación contra el SARS-CoV-2 y por el tipo de variante que predominó en cada ola de la pandemia ^5^.

Debido al cambio epidemiológico del SIM-C en los últimos años ^4^, es importante conocer como ha variado las características del SIM-C durante la pandemia en Perú, de esta manera mejorar los protocolos de atención para futuros escenarios y evitar complicaciones en los pacientes. El objetivo del trabajo fue describir las características del SIM-C en los primeros tres años de la pandemia, en un hospital pediátrico de Perú según la ola y el desarrollo de shock.

**MENSAJES CLAVE**

Motivación para realizar el estudio. Existen limitados estudios que describan la variación del síndrome inflamatorio multisistémico asociado a COVID-19 (SIM-C) en Perú a través de las olas de la pandemia.

Principales hallazgos. En los primeros tres años de pandemia los casos de SIM-C disminuyeron, con mayor la frecuencia en la segunda ola con características clínicas similares a la enfermedad de Kawasaki.

Implicancias. El SIM-C es una complicación posinfecciosa del SARS-CoV-2. Es importante su sospecha diagnóstica semanas posteriores a los picos de contagios, especialmente en los niños que aún no han recibido vacunas contra la COVID-19.

## EL ESTUDIO

Se realizó un estudio observacional, descriptivo y retrospectivo. La población de estudio estuvo conformada por los pacientes hospitalizados con el diagnóstico de SIM-C entre abril del 2020 hasta diciembre del 2022 en el Instituto Nacional de Salud del Niño-Breña (INSN-B) de Lima, Perú. El INSN-B, es uno de los hospitales pediátricos con mayor capacidad resolutiva ubicado en Lima, cuenta con distintas especialidades y subespecialidades dentro de ellas la unidad de cuidados intensivos pediátricos (UCIP) para los pacientes críticos. El INSN-B, es una institución estatal que pertenece al Ministerio de Salud de Perú (MINSA) y atiende a la población con o sin seguro integral de salud (SIS) siendo una de las instituciones con mayor representatividad en Perú, tanto asistencial como de investigación ^16^.

 El diagnóstico de SIM-C se realizó en base a los criterios de la Organización Mundial de la Salud (OMS) ^1^, similar a reportes previos ^12^^,^^13^. Se incluyeron los pacientes con diagnóstico de SIM-C al ingreso por emergencia o durante la hospitalización, Fueron excluidos aquellos con infección por SARS-CoV-2 con criterios de EK sin criterios de SIM-C (tres pacientes), aquellos diagnosticados de otra enfermedad (uno con púrpura trombocitopénica trombótica, uno con arteritis de Takayasu y dos con dengue) y aquellos referidos a otro hospital (tres pacientes).

No se realizó un cálculo de tamaño de muestra debido a los limitados casos pues es una enfermedad poco frecuente. La recolección de datos fue realizado por cuatro investigadores distribuidos en grupos de 2 de manera independiente. Las discrepancias detectadas en la base de datos fueron resueltas por un quinto investigador realizando nuevamente la revisión.

### Variables de estudio

Las variables del estudio fueron la edad (años), sexo (masculino y femenino), contacto intradomiciliario con persona infectada por SARS-COV-2 (sí y no), tipo de prueba SARS-CoV-2: reacción en cadena de polimerasa en tiempo real (RT-PCR), antigénica y serológicas (IgM e IgG), comorbilidades, tiempo de enfermedad (días), sintomatología gastrointestinal (dolor abdominal, vómitos, diarrea, náuseas), mucocutáneas (exantema polimorfo, inyección conjuntival, cambios en la cavidad oral, edemas de palmas y plantas, descamación de los dedos y linfoadenopatías cervicales), respiratorias (síntomas respiratorios altos, dificultad respiratoria), neurológicas y el fenotipo clínico (fiebre e inflamación, similar a EK y shock), basado en reportes previos ^6^^,^^17^^,^^18^.

También se consideraron las características laboratoriales, tratamiento (inmunoglobulina, corticoide, ácido acetil salicílico, vasopresores/inotrópicos, anticoagulación y antibióticos), complicaciones (alteraciones en la ecocardiografía, ingreso a UCIP y ventilación mecánica invasiva, síndrome de activación de macrófagos) y tiempo de hospitalización (días) (Tabla S1).

Las variables se describieron de forma general (periodo 2020-2022), según las primeras tres olas por COVID-19 (primera: marzo - diciembre 2020, segunda: enero - setiembre 2021, tercera: diciembre 2021 - enero 2022) ^19^ y según el fenotipo shock (SIM-C con necesidad de soporte con inotropo/vasopresor o reanimación de fluidos >20 ml/kg).

### Procedimiento y análisis estadístico

Se identificaron los registros de los pacientes incluidos en el estudio y luego se procedió a la revisión y recopilación de la información de sus historias clínicas del archivo del hospital. Se usó una ficha de recolección de datos, previa aprobación y autorización del proyecto por el comité de investigación institucional del hospital.

Para el análisis de datos se utilizó el programa STATA v.16 (StataCorp LP, College Station, Texas, Estados Unidos). Las variables categóricas se describieron mediante frecuencias absolutas y relativas y las cuantitativas, mediante media y desviación estándar (DE) o mediana y rango intercuartílico (RIC) según la evaluación de normalidad con la prueba Shapiro-Wilk.

### Aspectos éticos

El trabajo fue aprobado por el Comité de Ética en Investigación del INSN-B (N° 078-2022-CIEI-INSN) y el protocolo fue registrado en la plataforma PRISA del Instituto Nacional de Salud (código: EI00002522). No se solicitó consentimiento informado porque se optó por el permiso de la institución para la recolección de datos de las historias clínicas y se respetó la confidencialidad de los datos usando un código numérico. Algunas características de los primeros ocho pacientes del estudio fueron publicadas en la Revista Peruana de Medicina Experimental y Salud Pública ^20^.

## HALLAZGOS

De los 73 pacientes con SIM-C, cerca del 50% fueron diagnosticados en la segunda ola (Figura 1) y el último paciente diagnosticado en abril del 2022 (tercera ola). La mediana de edad fue 6 años (RIC: 2 - 9, rango: 3 meses - 16 años) y fue mayor en la segunda y tercera ola. Cinco (6,9%) tenían menos de un año, 23 (31,5%) uno a cuatro años, 32 (43,8%) cinco a nueve años y 13 (17,8%) mayor de 10 años. Más de la mitad fueron varones y 61 (83,6%) pacientes tenían contacto intradomiciliario con persona infectada por SARS-COV-2. Sesenta y uno (83,6%) tenía alguna prueba de SARS-CoV-2 positiva (6/59 RT-PCR, 3/49 antigénica, 17/56 IgM y 52/64 IgG) y 12 tuvieron pruebas negativas con contacto intradomiciliario positivo a SARS-CoV-2. Dos pacientes tenían comorbilidades (uno vejiga neurogénica y otra hidronefrosis unilateral). La mediana del tiempo de enfermedad fue 5 días (RIC: 4-7) y fue mayor en la tercera ola (Tabla 1).

**Figura 1:** Hospitalizaciones por síndrome inflamatorio multisistémico asociado a COVID-19 (SIM-C) según olas de la pandemia (primera ola: marzo-diciembre 2020, segunda ola: enero-setiembre 2021, tercera ola: diciembre 2021-enero 2022).

**Tabla 1:** Características generales y clínicas de pacientes con síndrome inflamatorio multisistémico asociado a COVID-19 (SIM-C).

Características		Total n=73 (%)	Olas de la pandemia			Shock	
			Primera ola n=29 (%)	Segunda ola n=35 (%)	Tercera ola n=9 (%)	No n=52 (%)	Sí n=21 (%)
Edad (años) ^a^		6 (2-9)	5 (2-7)	6 (2-9)	9 (1,6-13,8)	5 (2-8.5)	8 (5-9)
Sexo masculino		46 (63,0)	19 (65,5)	20 (57,1)	7 (77,8)	33 (63,5)	13 (61,9)
Contacto intradomiciliario		61 (83,6)	22 (78,9)	31 (88,6)	8 (88,9)	42 (80,8)	19 (90,5)
Tiempo de enfermedad (días) ^a^		5 (4-7)	5 (4-7)	5 (4-7)	6 (5-8)	5,5 (4-7)	5 (4-5)
Manifestaciones clínicas							
	Tiempo de fiebre (días) ^a^	5 (4-7)	4 (4-6)	5 (4-7)	6 (5-8)	5 (4-7)	5 (4-6)
	Gastrointestinales ^b^	60 (82,2)	23 (79,3)	29 (82,9)	8 (88,9)	42 (80,8)	18 (85,7)
	Dolor abdominal	41 (56,2)	16 (55,2)	22 (62,9)	3 (33,3)	25 (48,1)	16 (76,2)
	Vómitos	32 (43,8)	12 (41,3)	15 (42,9)	5 (55,6)	18 (40,0)	12 (57,1)
	Diarrea	30 (41,1)	10 (34,5)	16 (45,7)	4 (44,4)	22 (42,3)	8 (38,1)
	Náuseas	12 (16,4)	4 (13,8)	5 (14,3)	3 (33,3)	7 (13,5)	5 (23,8)
	Mucocutáneas ^b^	66 (90,4)	26 (89,7)	31 (88,6)	9 (100,0)	47 (90,4)	19 (90,5)
	Exantema polimorfo	55 (75,3)	21 (72,4)	26 (74,3)	8 (88,9)	40 (76,9)	15 (71,4)
	Inyección conjuntival	48 (65,8)	20 (69,0)	22 (62,9)	6 (66,7)	32 (61,5)	16 (76,2)
	Cambios en labios/lengua/mucosa oral	25 (34,3)	6 (20,7)	13 (37,1)	6 (66,7)	19 (36,5)	6 (28,6)
	Edema de palmas y plantas	23 (31,5)	7 (24,1)	12 (34,3)	4 (44,4)	18 (34,6)	5 (23,8)
	Descamación de los pulpejos de los dedos	5 (6,9)	0 (0,0)	4 (11,4)	1 (11,1)	2 (3,9)	3 (14,3)
	Linfoadenopatías cervicales >1,5 cm	13 (17,8)	4 (13,8)	7 (20,0)	2 (22,2)	7 (13,5)	6 (28,6)
	Respiratorias	21 (28,8)	6 (20,7)	11 (31,4)	4 (44,4)	14 (26,9)	7 (33,3)
	Síntomas respiratorios altos (tos, coriza, odinofagia)	14 (19,2)	4 (13,8)	7 (20,0)	3 (33,3)	12 (23,1)	2 (9,5)
	Dificultad respiratoria (taquipnea, tiraje, quejido)	9 (12,3)	2 (6,9)	5 (14,3)	2 (22,2)	3 (5,8)	6 (28,6)
	Neurológicas (cefalea, irritabilidad, convulsiones)	14 (19,2)	3 (10,3)	9 (25,7)	2 (22,2)	6 (11,5)	8 (38,1)
Fenotipo clínico							
	Fiebre e inflamación	18 (24,6)	5 (17,2)	9 (25,7)	4 (44,4)	--	--
	Similar a enfermedad de Kawasaki	34 (46,6)	16 (55,2)	14 (40,0)	4 (44,4)	--	--
	Shock	21 (28,8)	8 (27,6)	12 (34,3)	1 (11,1)	--	--

a Mediana y rangos intercuartiles, ^b^ presentaban más de un síntoma. Shock: Necesidad de soporte inotrópico o reanimación con líquidos >20 mL/kg.

Las manifestaciones gastrointestinales (dolor abdominal y vómitos) y mucocutáneas (exantema polimorfo e inyección conjuntival) fueron frecuentes en las tres olas, y las respiratorias y neurológicas en la segunda ola. El fenotipo similar a la EK fue frecuente en las tres olas y se reportó en 34 (46,6%) pacientes seguido del fenotipo shock en 21 (28,8%) pacientes.

En la analítica sanguínea se encontró anemia leve 10,5 g/dL (RIC: 9,7-11,2), y elevación de leucocitos 12500/mm^3^ (RIC: 7330-16390), neutrófilos 8772/mm3 (RIC: 5057,7-12002), velocidad de sedimentación globular 39,5 mm/h (RIC: 27,5-49), proteína C reactiva 18,2 mg/dL (RIC: 11,8-24,8), enzimas hepáticas, fibrinógeno 558 mg/dL (RIC: 493,3-651,5), dímero D 4,2 mg/L (RIC: 2,3-7,1), ferritina 402 ng/mL (RIC: 234,5-538,5) y lactato deshidrogenasa 547 U/L (RIC: 432-665) (Tabla 2).

**Tabla 2:** Características laboratoriales de pacientes con síndrome inflamatorio multisistémico asociado a COVID-19 (SIM-C).

Características laboratoriales	Total n=73	Shock	
		No n=52	Sí n=21
	Mediana (RIC)	Mediana (RIC)	Mediana (RIC)
Hemoglobina (g/dL)	10,5 (9,7-11,2)	10,7 (9,7-11,2)	10,2 (9,7-11,4)
Leucocitos (cel/mm^3^)	12500 (7330-16390)	13170 (8315-16420)	10260 (6100-14760)
Neutrófilos (cel/mm^3^)	8772 (5057,7-12002)	9618,8 (4975-12334,9)	8174 (5322,2-11219)
Linfocitos (cel/mm^3^)	1979,1 (893,6-3452)	2332,6 (1073,1-4461,3)	1405,2 (542,3-2902,6)
Eosinófilos (cel/mm^3^)	73,3 (0-285,4)	95,4 (0-286,6)	59,8 (35-237,7)
Plaquetas (cel/mm^3^)	17400 (110000-323000)	196000 (116000-369500)	138000 (100000-252000)
Velocidad de sedimentación globular (mm/h)	39,5 (27,5-49)	40 (25-50)	38,5 (32-45)
Proteína c reactiva (mg/dL)	18,2 (11,8-24,8)	17,7 (10,9-24,5)	18,5 (13,6-27,3)
Aspartato aminotrasnferasa (UI/L)	44 (34-67)	43 (33-74)	44 (42-49)
Alanino aminotransferasa (UI/L)	47,5 (23-77)	43 (20-77)	50,5 (27-120)
Gamma-glutamiltransferasa (UI/L)	54,2 (27-176,5)	38 (19-86)	175 (59-301)
Fosfatasa alcalina (UI/L)	415 (306,5-504,5)	375,5 (286-466,5)	458,5 (405,5-623,5)
Fibrinógeno (mg/dL)	558 (493,3-651,5)	558 (509,8-658,5)	557 (492,2-643,4)
Dímero D (mg/L)	4,2 (2,3-7,1)	3,9 (2,3-7,2)	4,5 (2,4-6,9)
Albúmina (g/dL)	3,7 (3,3-4)	3,7 (3,4-3,9)	3,4 (3,1-4)
Sodio (mmol/L)	135 (132-137)	134 (131-136)	136,5 (132,5-138,5)
Potasio (mmol/L)	3,9 (3,6-4,3)	3,9 (3,7-4,4)	3,9 (3,6-4,2)
Ferritina (ng/mL)	402 (234,5-538,5)	359 (217-529)	436 (370-649)
CPK-MB (UI/L)	20 (15,5-43)	21,5 (13,5-29,5)	19,5 (17-29)
Lactato deshidrogenasa (UI/L)	547 (432-665)	560 (458-737)	462 (426-644)
Urea (mg/dL)	27 (19-40)	23 (18-40)	34 (21-40)
Creatinina (mg/dL)	0,5 (0,4-0,6)	0,5 (0,4-0,6)	0,5 (0,4-0,5)

RIC: rango intercuartílico, CPK-MB: creatinofosfoquinasa miocárdica, shock: necesidad de soporte inotrópico o reanimación con líquidos >20mL/kg

La inmunoglobulina humana endovenosa se utilizó en 70 (95,9%) pacientes con una dosis de 1 a 2gr/kg (mínimo: 9 gramos; máximo: 100 gramos) y fue administrado en todos los pacientes en la primera ola. El corticoide (metilprednisolona) fue utilizado con mayor frecuencia en la segunda ola en 34 (97,1%) pacientes. La dosis utilizada fue 1-2mg/kg por cinco días, y como dosis de pulso 10-30mg/kg (14 pacientes) en aquellos con signos de deterioro clínico por tres días. El ácido acetil salicílico se utilizó en 69 (94,5%) pacientes, 47 (64,4%) recibieron anticoagulación profiláctica y 57 (78,1%) antibióticos, estos dos últimos disminuyeron su uso en cada ola.

Como complicaciones, cinco desarrollaron aneurisma coronario y tres, disfunción ventricular izquierda. Dos pacientes requirieron intubación en la emergencia, 17 (23,3%) ingresaron a UCIP (media 5,8+/-3 días) y 11 (15,1%) requirieron ventilación mecánica invasiva (media 4+/-1 día). Diez (15,1%) pacientes tuvieron síndrome de activación de macrófagos, seis (8,2%) neumonía, dos pancreatitis y uno, trombosis venosa profunda en las extremidades. La mediana del tiempo de hospitalización fue 10 días (RIC: 8-14) y ningún paciente falleció durante la hospitalización.

Los pacientes con fenotipo shock tuvieron una mayor edad con una mediana de ocho años (RIQ 5-9), mayor dificultad respiratoria y sintomatología neurológica en comparación con aquellos que no desarrollaron shock (Tabla 1). Además, en este grupo de pacientes se observó mayor alteración inflamatoria (incremento de la proteína c reactiva, y ferritina), alteración de las enzimas hepáticas, alteración hematológica (menor hemoglobina, linfopenia y trombocitopenia leve) y alteración de la coagulación (fibrinógeno y dímero D) (Tabla 2). El uso de segunda dosis de inmunoglobulina fue mayor (47,6%) y todos usaron vasopresores y antibióticos (Tabla 3). Las alteraciones ecocardiográficas fueron más frecuentes en estos pacientes y tuvieron mayor tiempo de hospitalización, 14 días (RIC: 11-16).

**Tabla 3:** Tratamiento y complicaciones en pacientes con síndrome inflamatorio multisistémico asociado a COVID-19 (SIM-C).

Características			Total n=73 (%)	Ola de COVID-19			Shock	
				1era Ola n=29 (%)	2da Ola n=35 (%)	3era Ola n=9 (%)	No n=52 (%)	Si n=21 (%)
Tratamiento								
	Inmunoglobulina humana (1era dosis)		70 (95,9)	29 (100,0)	33 (94,3)	8 (88,9)	49 (94,2)	21 (100,0)
	Inmunoglobulina humana (2da dosis)		19 (26,0)	7 (24,1)	11 (31,4)	1 (11,1)	9 (17,3)	10 (47,6)
	Corticoide		63 (86,3)	21 (72,4)	34 (97,1)	8 (88,9)	44 (84,6)	19 (90,5)
	Ácido acetil salicílico		69 (94,5)	27 (93,1)	34 (97,1)	8 (88,9)	50 (96,2)	19 (90,5)
	Vasopresores/inotrópicos		21 (28,8)	8 (27,6)	12 (34,3)	1 (11,1)	0 (0,0)	21 (100,0)
	Anticoagulación							
	Tratamiento		5 (6,9)	2 (6,9)	2 (5,7)	1 (11,1)	1 (1,9)	4 (19,1)
	Profiláctico		47 (64,4)	20 (69,0)	23 (65,7)	4 (44,4)	32 (61,5)	15 (71,4)
	Antibióticos		57 (78,1)	24 (82,8)	28 (80,0)	5 (55,6)	36 (69,2)	21 (100,0)
Complicaciones								
	Ecocardiografía (n=71)^a^							
		Aneurisma coronario	5 (7,0)	1 (3,6)	3 (8,6)	1 (12,5)	3 (5,9)	2 (10,0)
		Disfunción ventricular izquierda	3 (4,2)	2 (7,1)	1 (2,9)	0 (0,0)	1 (2,0)	2 (10,0)
		Efusión pericárdica	7 (9,9)	5 (17,9)	1 (2,9)	1 (12,5)	3 (5,9)	4 (20,0)
		Alteración valvular	4 (5,6)	3 (10,7)	1 (2,9)	0 (0,0)	1 (2,0)	3 (15,0)
		Dilatación ventricular	1 (1,4)	1 (3,6)	0 (0,0)	0 (0,0)	0 (0,0)	1 (5,0)
	Ingreso a UCIP		17 (23,3)	7 (24,1)	9 (25,7)	1 (11,1)	0 (0,0)	17 (80,9)
	Ventilación mecánica invasiva		11 (15,1)	4 (13,8)	6 (17,1)	1 (11,1)	0 (0,0)	11 (52,4)
	Síndrome de activación de macrófagos		10 (15,1)	4 (13,8)	5 (14,3)	2 (22,2)	8 (15,4)	3 (14,3)
	Neumonía		6 (8,2)	2 (6,9)	4 (11,4)	0 (0,0)	3 (5,8)	3 (14,3)
	Tiempo de hospitalización (días) ^b^		10 (8-14)	10 (7-13)	10 (8-14)	8 (8-10)	8,5 (7-11)	14 (11-16)

a Presentaban más de un hallazgo, ^b^ mediana (RIC).

RIC: rango intercuartílico, UCIP: Unidad de Cuidados Intensivos Pediátricos, shock: necesidad de soporte inotrópico o reanimación con líquidos > 20mL/kg.

## DISCUSIÓN

En nuestro estudio se observó que la frecuencia de SIM-C fue variando en los primeros tres años de la pandemia y se caracterizó por sintomatología gastrointestinal y mucocutánea. El tratamiento consistió en el uso de inmunoglobulina y corticoides, y aquellos con shock tuvieron mayores complicaciones.

Encontramos más casos diagnosticados en la segunda ola similar a lo reportado en un hospital de India ^13^ pero en Francia se reportaron más casos en la primera ola ^12^. Esta diferencia se podría explicar porque en Perú el pico elevado de contagios ocurrió en la segunda ola adicionado al ablandamiento de las medidas de distanciamiento social por la reactivación económica y mayor circulación de personas entre ellos los niños ^19^. El SIM-C, se ha reportado después de cuatro a seis semanas del pico de contagios por COVID-19 ^1^, y esto guarda relación con lo reportado en Francia en su primera ola ^12^.

Las variantes también pueden influir en la frecuencia de casos de SIM-C. En Perú, la identificación de las variantes inició en la segunda ola (2021) siendo predominante la variante Lambda/Gamma/Delta, y en la tercera ola, la variante Ómicron ^19^ donde se registraron menos SIM-C a pesar de un pico elevado de contagios. La vacunación pudo influir en la disminución de casos de SIM-C durante la tercera ola. Este efecto de la vacunación se reportó durante la ola por la variante Delta en niños de 12 a 18 años en Estados Unidos ^21^, y durante la ola por la variante Ómicron en Dinamarca ^5^.

En Perú, la vacunación (Pfizer-BioNTech) se inició en niños de 12-17 años en noviembre del 2021 y de 5-11 años en enero del 2022. En nuestro estudio dos pacientes con SIM-C habían recibido vacunación contra COVID-19, un varón de 14 años (vacunado en noviembre 2021, una dosis) y desarrolló la enfermedad cinco meses después (tenía IgG positivo), y otro varón de 11 años (vacunado en febrero 2022) y desarrolló la enfermedad ocho días después, pero tenía serología IgG e IgM positivo al diagnóstico.

La mediana de edad fue de seis años, menor a lo reportado en otros estudios ^9^^,^^18^ y mayor en las posteriores olas, tendencia similar a lo reportado en Argentina ^14^. Las manifestaciones gastrointestinales y mucocutáneas fueron las más frecuentes concordante con otros estudios ^12^^,^^13^. Estas características fueron reportadas desde los primeros casos en Reino Unido ^6^, y actualmente se mantiene en reportes en Dinamarca ^5^, Estados Unidos ^11^ y Latinoamérica ^3^^,^^10^.

Los niños con SIM-C desarrollan alteraciones laboratoriales como elevación de marcadores inflamatorios, trastornos hematológicos y de coagulación, esto fue visto en los pacientes especialmente en aquellos con fenotipo shock concordante con reportes previos ^9^^,^^18^. Estas alteraciones se producen por un desequilibrio en el sistema inmunitario producto de la unión del SARS-CoV-2 a los receptores ACE-2 que están en las células endoteliales del sistema vascular provocando inflamación y coagulación sistémica ^1^.

El tratamiento consistió en el uso de la inmunoglobulina, corticoide y ácido acetil salicílico, similar al manejo de la EK ^22^ y de acuerdo a algunos consensos de manejo de SIM-C de países de Latinoamérica como Chile ^17^^)^ y Argentina ^15^. Algunos pacientes requirieron una segunda dosis de inmunoglobulina y fueron aquellos que no presentaban mejoramiento clínico después de 36 horas de administrada la primera dosis, tenían persistencia de fiebre o desarrollaban shock o síndrome de activación de macrófagos.

El uso de antibióticos fue elevado a inicios de la pandemia y fueron los pacientes con SIM-C o COVID-19 severo quienes tuvieron más riesgo de recibirla ^18^^,^^20^. En nuestro estudio, los antibióticos fueron más usados durante la primera y segunda ola por el riesgo de infección bacteriana; sin embargo, fue menor en la tercera ola, tendencia similar a lo reportado en Francia ^12^. Esto se explica porque a medida que pasaban las olas, se realizaba un mejor diagnóstico y tratamiento evitando el uso de antibióticos por ser el SIM-C una complicación viral posinfecciosa.

 Las complicaciones cardiacas es lo que caracteriza al SIM-C en el fase inicial ^23^^,^^24^. En nuestro estudio, cinco (7,0%) pacientes desarrollaron aneurisma coronario y tres (4,2%), disfunción ventricular izquierda, ninguno tuvo dilatación de arterias coronarias. Esta baja frecuencia puede explicarse porque hubo menos casos severos que resultó en menor ingreso a UCIP y necesidad de ventilación mecánica a diferencia de otras series ^3^^,^^12^^,^^13^^,^^23^^,^^24^; sin embargo, aquellos pacientes con shock tuvieron más alteraciones cardiacas y complicaciones similar que otros reportes ^9^^,^^11^^,^^18^^,^^24^. Ningún paciente falleció durante la hospitalización pero en otros estudios se ha reportado una letalidad entre el 1 al 9% ^9^^,^^13^^,^^14^^,^^18^.

El estudio tuvo limitaciones por ser de tipo retrospectivo y por realizarse en un solo hospital, por lo que estos hallazgos no se podrían generalizar a otras instituciones del Perú. No se pudo identificar el tipo de variante de SARS-CoV-2 en cada paciente, lo que hubiese servido para caracterizar mejor a cada grupo; sin embargo, los casos tenían relación temporal con las olas de la COVID-19. A pesar de ello, el estudio tiene la fortaleza de describir características de una considerable serie de casos de SIM-C de un hospital pediátrico de referencia nacional durante los primeros tres años de la pandemia, donde el sistema de salud peruano tuvo limitados recursos humanos, materiales y de logística.

En conclusión, en el INSN-B la frecuencia de casos de SIM-C varió en los primeros tres años de la pandemia. Las manifestaciones clínicas y el tratamiento fueron similares a lo descrito en otros estudios y los pacientes con shock tuvieron mayor alteración laboratorial y complicaciones cardiacas.
